# Isolated Epitrochlear Filarial Lymphadenopathy: Cytomorphological Diagnosis of an Unusual Presentation

**DOI:** 10.5146/tjpath.2018.01423

**Published:** 2020-01-15

**Authors:** Priya Sahu, Prajwala Gupta, Minakshi Bhardwaj, CK Durga

**Affiliations:** Department of Pathology, Post Graduate Institute of Medical Education and Research, Dr. Ram Manohar Lohia Hospital, New Delhi, India; Department of Post Graduate Institute of Medical Education and Research, Dr. Ram Manohar Lohia Hospital, New Delhi, India

**Keywords:** Filariasis, Microfilaria, Lymph node, Cytology

## Abstract

Filariasis is a major public health problem in tropical countries like India. Despite the large number of people at risk, detection of eggs with or without larva (microfilaria) on fine-needle aspiration cytology is very unusual, especially in an uncommon site or incidentally detected in clinically unsuspected cases of filariasis with the absence of microfilariae in the peripheral blood. A 19-year-old male presented with swelling over medial aspect of left arm (just above the elbow), with no other specific signs and symptoms. Fine needle aspiration cytology revealed an adult gravid female filarial worm in a background of reactive lymphoid cells and lymphohistiocytic clusters. We report a case with elaborate fine needle aspiration cytology findings of filarial worm infestation with unusual presentation of isolated epitrochlear lymph node involvement in a clinically unsuspected case and recommend clinicians and pathologists to consider a high index of suspicion for such infections at uncommon sites especially in endemic territories, as early diagnosis and treatment prevent the more severe manifestations of disease.

## INTRODUCTION

Filariasis has been a major public health problem in tropical and subtropical countries (1). Microfilaria have been reported at various sites involving lymphatic systems and subcutaneous tissue, though meagre cases have been reported of filarial infestation with epitrochlear lymph nodes involvement (2).

Lymphatic vessels of the definitive host (man) harbours the adult worms of *Wuchereria* *bancrofti* and microfilaria are released in peripheral blood (3). However, all components of filarial worm infestation such as adult filarial worm with eggs and microfilaria in varying stages are rarely found together on fine needle aspiration cytology (FNAC) of lymph node swellings. We present one such elaborate cytomorphological findings of filarial worm infestation in a case with isolated involvement of an epitrochlear lymph node.

## CASE REPORT

**Figure 1:**
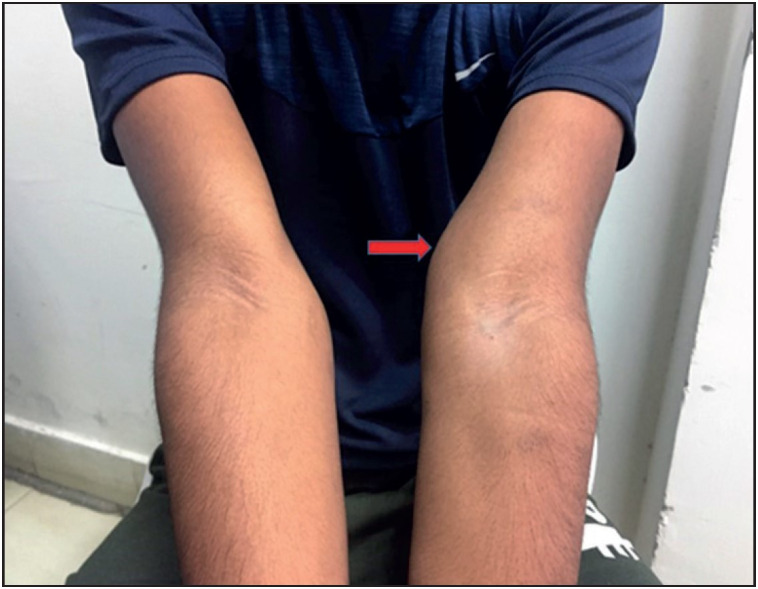
Swelling over medial aspect of left arm above elbow (red arrow).

**Figure 2:**
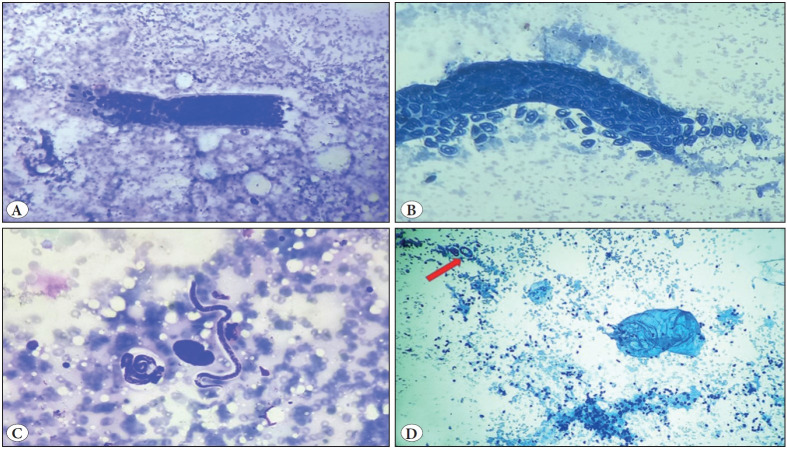
**A)** Adult gravid segment of filarial worm packed with embryonated eggs. (MGG; x100). **B)** Adult filarial worm segment with coiled forms of microfilaria (PAP; x100). **C)** Embryonated egg, coiled and uncoiled forms of sheathed microfilaria (MGG; x400). **D)** Focally the smears had lymphocytes and a lymphohistiocytic cluster and the arrow shows coiled microfilaria. (PAP; x40).

A 19-year-old male, resident of Bihar, presented to the FNA clinic with complaints of a painless swellings over the medial aspect of the left arm just above the elbow for 8 months, which had gradually increased in size. He had no complaints of fever or any other swelling in the body. General examination was normal and did not reveal any palpable peripheral lymphadenopathy. On local examination there were two swellings, located superficially on the medial aspect of the left arm just above the elbow, measuring 2x2cm and 1x1cm (Figure 1). The swellings were firm, mobile and non-tender with normal appearing overlying skin. FNAC was done and the air dried and alcohol fixed slides were stained with Giemsa and Papanicoloau stain respectively. Cytopathological examination of these slides revealed gravid segments of adult filarial worm and embryonated eggs (Figure 2) with various stages of microfilaria (coiled and uncoiled) (Figure 2). The microfilaria seen were sheathed with absence of nuclei at tip (*W. bancrofti*) (Figure 2). Background was haemorrhagic and showed focal reactive lymphoid cells and lymphohistiocytic clusters. (Figure 2) On FNAC, a diagnosis of parasitic infestation with filarial lymphadenopathy was made. Complete blood count revealed Hb.-13.2g/dL, TLC- 4700/mm3 and differential count revealing eosinophilia (14%) with absolute eosinophil count of 648/mm3 (normal range- 30-350/mm3). Peripheral blood did not reveal microfilaria. The patient was started on Diethylcarbamazine citrate (DEC) and on follow up the swelling was reduced.

## DISCUSSION

Filariasis is an endemic problem in many parts of India such as Assam, West Bengal, Bihar, Odisha, Uttar Pradesh, Madhya Pradesh, Tamil Nadu and Jharkhand (4). In endemic communities, filariasis is usually asymptomatic and symptoms are caused by progressive lymphatic vascular dilatation (1). Humans are the definitive host and infection is transmitted by mosquitoes. It can cause lymphatic filariasis or non-lymphatic filariasis involving subcutaneous tissue or serous cavities. Lymphatic filariasis represents about 25-30% of all cases of filariasis, caused most commonly by *Wuchereria bancrofti*, followed by *Brugia malayi* or *Brugia timori* (5). These worms occupy the lymphatic system, including the lymph nodes (6). Serous cavity filariasis is caused by *Mansonella* species. In cases of lymphatic filariasis, it has a predilection for lymphatic system of the lower limbs and male genitalia, producing episodic funiculitis, epididymitis and orchitis (7). Filariasis accounts for 0.047% of all cases of lymph node swellings with a predilection for inguinal and axillary lymph nodes and causes retrograde lymphangitis (5,7,8).

Microfilariae have been incidentally detected and reported in fine needle aspirates of various visceral sites like the breast, thyroid, spleen, ovarian fluid and bone marrow. Rare reports of microfilaria have been noted in the urine and in an upper arm cystic swelling in clinically unsuspected cases of filariasis with the absence of microfilariae in the peripheral blood (1,8,9).

Epitrochlear lymph nodes may be enlarged as a part of generalized lymphadenopathy and isolated enlargement is seldom seen. Tuberculosis, cat scratch disease, leprosy and leishmaniasis are a few reported benign causes of isolated epitrochlear lymph node enlargement; malignant causes include lymphoma and malignant melanoma (2). After extensive literature search, meagre reported cases were found of filarial involvement of epitrochlear lymph nodes (2,10).

Our case is an addition to unusual sites of filarial involvement as an isolated epitrochlear lymph node enlargement. Moreover in the present case, all the components of filarial worm infestation consisting of gravid female worm with eggs and different stages of microfilaria were demonstrated on FNAC.

We reiterate that the demonstration and identification of different parasitic components in cytology smears play a significant role in early diagnosis of the disease. This would prompt the clinicians for instituting a specific treatment and consider a high index of suspicion for filariasis at uncommon sites especially in endemic areas.

## Conflict of Interest

The authors declare that they have no competing interest.
